# Jinhua Qinggan granules for non-hospitalized COVID-19 patients: A double-blind, placebo-controlled, and randomized controlled trial

**DOI:** 10.3389/fmed.2022.928468

**Published:** 2022-08-01

**Authors:** Muhammad Raza Shah, Samreen Fatima, Sehrosh Naz Khan, Shafi Ullah, Gulshan Himani, Kelvin Wan, Timothy Lin, Johnson Y. N. Lau, Qingquan Liu, Dennis S. C. Lam

**Affiliations:** ^1^Center for Bioequivalence Studies and Clinical Research, Dr. Panjwani Center for Molecular Medicine and Drug Research, International Center for Chemical and Biological Sciences, University of Karachi, Karachi, Pakistan; ^2^The Indus Hospital Karachi, Karachi, Pakistan; ^3^School of Chinese Medicine of Hong Kong, Baptist University, Kowloon, Hong Kong SAR, China; ^4^Beijing Hospital of Traditional Chinese Medicine, Capital Medical University, Beijing, China

**Keywords:** Chinese mineral medicine, Traditional Chinese Medicine, Jinhua Qinggan granules (JHQG), COVID-19, randomized controlled (clinical) trial

## Abstract

**Background:**

Key findings from the World Health Organization Expert Meeting on Evaluation of Traditional Chinese Medicine (TCM) in treating coronavirus disease 2019 (COVID-19) reported that TCMs are beneficial, particularly for mild-to-moderate cases. The efficacy of Jinhua Qinggan granules (JHQG) in COVID-19 patients with mild symptoms has yet to be clearly defined.

**Methods:**

We conducted a phase 2/3, double-blind, randomized, placebo-controlled trial to evaluate the efficacy and safety of treatment with JHQG in mild, non-hospitalized, laboratory-confirmed COVID-19 patients. Participants were randomly assigned to receive 5 g/sacket of JHQG or placebo granules orally thrice daily for 10 days. The primary outcomes were the improvement in clinical symptoms and a proportion tested negative on viral polymerase chain reaction (PCR) after treatment. Secondary outcomes were the time to recover from clinical symptoms and changes in white blood cells (WBC) and acute phase reactants (C-reactive protein (CRP) and ferritin) on the 10th day after treatment initiation.

**Results:**

A total of 300 patients were randomly assigned to receive JHQG (150 patients) and placebo (150 patients). Baseline characteristics were similar in the two groups. In the modified intention-to-treat analysis, JHQG showed greater clinical efficacy (82.67%) on the 10th day of the trial compared with the placebo group (10.74%; rate difference: 71.93%; 95% CI 64.09–79.76). The proportion of patients with a negative PCR after treatment was comparable (rate difference: −4.67%; 95% CI −15.76 to 6.42). In contrast, all changes in WBC, ferritin, and CRP levels showed a statistically significant decline in JHQG (*P* ≤ 0.044) after treatment, but not the latter in placebo (*P* = 0.077). The median time to recovery of COVID-19-related symptoms including cough, sputum, sore throat, dyspnea, headache, nasal obstruction, fatigue, and myalgia was shorter in the JHQG group compared to the placebo group (*P* < 0.001 for all). Three patients experienced mild-to-moderate adverse events (AEs) duringthe treatment period in the JHQG group. Findings were similar between the modified intention-to-treat and the per-protocol analysis that included only patients who reported 100% adherence to the assigned regimen.

**Conclusion:**

Based on the time to recover from the COVID-19-related symptoms and AEs, it is concluded that JHQG is a safe and effective TCM for symptomatic relief of patients with mild COVID-19. A symptomatic improvement in the JHQG group patients was observed and JHQG use would have important public health implications in such patients.

**Clinical Trial Registration:**

The Trial was prospectively registered on www.clinicaltrials.gov with registration number: NCT04723524.

## Introduction

The coronavirus disease 2019 (COVID-19) caused by the severe acute respiratory syndrome coronavirus 2 (SARS-CoV-2) came under the attention of the international medical community when China first notified the World Health Organization (WHO) of a pneumonia outbreak of then-unknown etiology in December 2019 (1). COVID-19 was subsequently declared by the WHO as a Public Health Emergency of International Concern and further as a pandemic as SARS-CoV-2 infections surged globally (2, 3). With the advent of the highly contagious Omicron and potentially emerging novel variants, the COVID-19 pandemic remains a threat to public health worldwide (4). As of 4 April 2022, COVID-19 has resulted in over 490 million cases and more than 6.1 million deaths globally (5). Although various vaccines showed evidence of substantially reducing hospitalization and mortality, limited access and public hesitance to vaccination have hindered the attainment of herd immunity to halt the pandemic through vaccination (6, 7). Specific populations of patients, in particular, elderly patients and patients with chronic medical conditions (e.g., obesity, diabetes mellitus, malignancy, pulmonary diseases, and cardiovascular diseases), are at significantly heightened risk of progression to severe disease and mortality after SARS-CoV-2 infection (8, 9). Therefore, a demand for anti-COVID-19 treatment options which can prevent the progression to severe disease and mortality exists. Such therapeutic agents should be readily available to be administered to patients with mild COVID-19 at disease onset to prevent subsequent progression.

Currently, oral antiviral agents available under emergency use authorization by the Food and Drug Administration for COVID-19 include molnupiravir and nirmatrelvir (10). Recently, the WHO Expert Meeting on Evaluation of Traditional Chinese Medicine (TCM) in the treatment of COVID-19 has concluded that TCM is beneficial in mild-to-moderate COVID-19, as an add-on intervention to conventional treatment, TCM may shorten the time for viral clearance, resolution of clinical symptoms, and length of hospital stay (11). Various TCMs have been approved by China's National Administration of TCM to manage COVID-19 (12). In the *Diagnosis and Treatment Protocol for Novel Coronavirus Pneumonia (Trial Version 7)* released by China's National Health Commission & National Administration of TCM, Jinhua Qinggan granules (JHQG) was recommended as a treatment for fatigue and fever for patients with COVID-19 during the medical observation period (13).

Jinhua Qinggan granules is created from the two classical TCM formulae Ma Xing Shi Gan Decoction and Yin Qiao San Decoction (14), containing 11 herbs including *Honeysuckle, Ephedrae Herba, Armeniacae Semen Amarum, Scutellariae Radix, Forsythiae Fructus, Fritillariae thunbergii Bulbus, Anemarrhenae Rhizoma, Arctii Fructus, Artemisiae annuae Herba, Menthae haplocalycis Herba, Glycyrrhizae Radix et Rhizoma* along with a traditional Chinese mineral medicine, and Gypsum Fibrosum (15, 16). According to the past reports, JHQG has a great clinical impact on influenza with the symptoms of high fever, headache, aversion to cold, pharyngalgia, sneezing, cough, muscle ache, etc. JHQG was previously proposed as a potential TCM for the treatment of influenza and has been shown to shorten the duration of fever and recovery time in patients with influenza (17). In recent studies of JHQG in patients with COVID-19, JHQG was reported to increase the viral clearance rate in patients with COVID-19 as evidenced by an increased proportion of patients with negative nucleic acid tests after JHQG treatment (16). It has further been studied in a randomized controlled trial (RCT) which demonstrated that patients with COVID-19 treated with JHQG combined with Western medications (oseltamivir and arbidol) showed an increased improvement in fever and fatigue compared with patients treated with the antivirals alone (18). Nevertheless, the efficacy and safety of the independent use of JHQG in the treatment of patients with COVID-19 for the prevention of disease progression remain to be elucidated. Therefore, this clinical trial was aimed to evaluate the efficacy and safety of JHQG in non-hospitalized Pakistani COVID-19 patients with mild disease.

## Methods

This phase 2–3, double-blind, randomized, placebo-controlled clinical trial evaluated the efficacy and safety of JHQG among non-hospitalized COVID-19 adult Pakistani patients with mild symptoms. This study was approved by the Institutional Ethics Committee of the International Center for Chemical and Biological Sciences (ICCBS) and the Institutional Review Board of the Indus Hospital (Sector 39, Karachi, Sindh, Pakistan). The trial was prospectively registered on www.clinicaltrials.gov with registration number: NCT04723524. All study participants provided written informed consent before enrolling in the clinical trial.

### Eligibility

Eligible patients to be enrolled in this study fulfilled all of the following inclusion criteria: (1) age range of 18–75 years; (2) confirmed SARS-CoV-2 infection by real-time reverse transcription-polymerase chain reaction (RT-PCR); (3) mild symptom cases with a score of 2 or less having any of COVID-19-related fever, sore throat, cough, headache, malaise, nausea, vomiting, diarrhea, muscle pain, or loss of taste and smell symptoms; and (4) capable of providing written informed consent. Patients were excluded if they had any of the following: (1) previous confirmed SARS-CoV-2 infection; (2) moderate or critical COVID-19 infection with (a) respiratory failure and requiring mechanical ventilation, (b) shock, or (c) other organ failure requiring intensive care unit (ICU) support; (3) severe primary health conditions associated with cardiovascular, cerebrovascular, pulmonary, hepatic, renal, endocrine and hematological diseases, hematopoietic system (above grade II of cardiac function; alanine aminotransferase (ALT) and aspartate transaminase (AST) are 1.5 times higher than the normal value; creatinine above the upper limit of normal value), and mental illness or serious diseases affecting their survival, such as cancer or AIDS; (4) administered other antiviral, antibiotics, cough relieving, and antihistamine medications within 3 days prior to the visit (e.g., β2 receptor agonists, anticholinergic agents, theophylline, glucocorticoids, cough expectorant, and other TCM); (5) history of drug or food allergy; (6) pregnancy, lactating, or fertile women who were planning to conceive in 3 months; and (7) participated in another clinical study in the past 1 month. The patients' inclusion and enrollment in the trial were made by the clinical trial investigator, considering and reviewing the laboratory tests and patients' conditions as per the seven-category ordinal scale.

### Sample size calculation

To detect a 20% difference in recovery defined by PCR test negativity and asymptomatic clinical state (40% with placebo vs. 60% with TCM) at 90% power, 260 patients (130 per group) are required at *p* < 0.05. The 300-sample size will allow for around 15% follow-up loss without loss of statistical power.

### Drug under investigation

Jinhua Qinggan granules [Chinese medicine Z20160001; Ju Xie Chang (Beijing) Pharmaceuticals Co., Ltd. (Jingjintang Kejiyuan Zhengzhong, Daxing, Beijing, China)] having batch no. 20200601 containing 5 g JHQG or placebo per sachets were used in this study. JHQG is synthesized from the two TCM formulae, namely, Ma Xing Shi Gan Decoction and Yin Qiao San Decoction (14). Packaging, presentation, and other characteristics of the JHQG and placebo were kept similar to prevent any allocation bias and ensure blinding. The products were packed according to the regulations of the Chinese Pharmacopoeia (19) in terms of quality standards.

### Patient allocation and assessments

Eligible patients were randomly assigned in a 1:1 ratio to receive either JHQG (5 g/sachet) or matched placebo and administered thrice daily for 10 days. Patients and investigators in this trial were blinded to the treatment allocation until the completion of the study. Randomized allocation sequences were generated by the sponsor of the trial through the SAS Analytics Software (version 9.4) using the block randomization method with parameters as a 1:1 arm ratio for JHQG and placebo; Block = 90; Rand = 4; and Seed = 2,020,068. The test drugs, i.e., JHQG and placebo were packed in similar dispensing boxes and pre-coded according to the randomization list by the sponsor to ensure the concealment of sequence and subjects were assigned to the group randomly. The study drugs were dispensed to the patients strictly according to the number mentioned on the investigational drugs' box. The investigational medicines were kept in contract research organization (CRO) under the supervision of a qualified pharmacist. These medicines were dispensed to the patients from the depot pharmacy of the CRO under the supervision of a clinical investigator. Data analysis was performed independently by professional statisticians to guarantee that all enrolled participants were evenly allocated to the JHQG or placebo groups. In case of severe adverse events (SAEs) or other unwanted situations, urgent unblinding permission was granted under the principal investigator (PI). Eligible patients received JHQG or placebo (5 g/sachet) at an oral dose of 5 g (1 sachet) three times a day after a meal dissolved in boiled water for 10 days. The course of treatment was 10 days, and the visit on the 10th day of treatment was set as a follow-up (Supplementary Table 1). All patients were reviewed on daily basis by investigators *via* telephone calls for medication consumption, health status, and patient diary record through day 10. The detailed assessment schedule is outlined in Supplementary Table 1.

### Study endpoints

Eligible patients were assessed using a seven-category ordinal scale (Supplementary Table 2) before the trial to include patients with score levels 1 and/or 2. Clinical signs and symptoms of the patients were graded using symptomatic grading criteria (Supplementary Table 3) on the 1st and 10th day of the trial period and recorded. All effectiveness and safety inspection items were done once before the trial and once at follow-up, i.e., 10th day. Demographics, vital signs, clinical symptoms, medication status, and AEs were recorded to evaluate the participants' degree of COVID-19-related symptom improvement (efficacy) according to the schedule given in Supplementary Table 1. Routine laboratory blood and urinary tests, electrocardiogram (ECG), serum electrolytes, liver function, renal function, creatine phosphokinase, SARS-CoV-2 RT-PCR, C-reactive protein (CRP), ferritin, and chest X-ray examination were performed at screening and 10th day of the trial to detect abnormal changes and assess the safety of the drug.

#### Efficacy endpoints

The primary efficacy endpoints of the study were (i) the improvement in main clinical signs and symptoms (cough and fever) based on the symptom grading scale (Supplementary Table 3) obtained on the 1st and 10th day after treatment initiation; and (ii) the proportion of patients who tested negative to nasopharyngeal SARS-CoV-2 test on RT-PCR at 10th day of treatment. Clinical efficacy was determined based on the combined scores of main symptom (MS) and secondary symptom (SS) and divided into “effective” including clinically cured, remarkable effective, and effective or “in-effective” including ineffective and worsened cases. Secondary efficacy endpoints were based on the documentation of body temperature; change in white blood cells (WBCs), CRP, and ferritin levels; change in radiographic (chest X-ray) findings; time to recovery of individual symptom; and quality of life assessment (given in Supplementary Material) at day 1 and 10th day after treatment initiation. The MS for improvement assessment were cough and fever, while sputum, sore throat, dyspnea, headache, nasal obstruction, myalgia, runny nose, chest pain, and diarrhea were SS. The MS score included cough and fever with 0, 2, 4, and 6 scales for no cough/no fever, intermittent cough/fever with body temperature of 98–100.4°F, cough mildly impacting the daily work/fever with body temperature of 100.4–102.2°F, and frequent cough or paroxysmal cough seriously impacting the work and life/fever with body temperature of 102.2–104°F, respectively. Similarly, the SS were sputum, sore throat, dyspnea, headache, nasal obstruction, myalgia, runny nose, chest pain, and diarrhea with 0 and 2 score levels for No and Yes (Supplementary Table 3). Each subject's score of MS (i.e., cough and fever) and SS as well as total scores (TS) were described and compared at baseline and at follow-up for JHQG and placebo groups. The recovery time from individual COVID-19-related symptoms, i.e., cough, sputum, sore throat, dyspnea, headache, nasal obstruction, myalgia, runny nose, chest pain, and diarrhea was analyzed using Kaplan–Meier to calculate the median time followed by log-rank to check the differences between the JHQG and placebo groups. The clinical efficacy of JHQG was judged based on the effectiveness of the drug in terms of before and after treatment scores of MS and SS (Supplementary Table 3).

The clinical efficacy was measured in terms of curative effect as “Yes” for remarkable effective (cough and fever are remarkably improved, two levels, i.e., lighten with a score of 6 → 2, and other SS are improved 2 → 0); effective (cough and fever are improved, lighten with scores of 6 → 4 and 4 → 2), and “No” for ineffective (no improvement of cough, same before and after the treatment). In addition, the curative effect of JHQG post-treatment was analyzed for each patient through curative index analysis for combined MS and SS grade of the patient using the following formula:


$$\begin{array}{l} Curative\;index\\ =\frac{Grade\;before\;treatment-\;Grade\;after\;treatment}{Grade\;before\;treatment}\times 100 \end{array}$$

Based on the curative index analysis of each patient, they were declared as clinically cured (symptom grade decreased ≥90%), remarkable effective (symptom grade decreased ≥70% and <90%), effective (symptom grade decreased ≥30% and <70%), ineffective (symptom grade decreased ≥0% and <30%), and worsened (symptom grade increased <0%). The radiologist who analyzed and graded the chest X-ray of the patients was kept blinded.

#### Safety

Safety endpoints included various clinical investigation results obtained from patients during the study period, namely, blood pressure, heart rate, respiration rate, ECG, chest X-ray at screening, and follow-up visits. Routine blood tests, urinalysis, serum electrolytes, liver function tests (ALT, AST, Total bilirubin (TBIL), Alkaline Phosphatase (AKP), Gamma-glutamyl Transferase (γ-GT)), and renal function tests [Blood urea nitrogen (BUN) and Cr] of patients in both groups at 0th and 10th days after treatment were performed. Medication compliance and AEs were assessed for all patients on follow-up (10th day). The rate of disease aggravation on the 10th day of treatment was also evaluated. All patients actively recorded any AEs potentially related to treatment with details including their occurrence, remission, and severity in patient diaries which were transcribed into detailed case report forms (CRFs) for investigators' review. The AEs were classified into mild AEs (asymptomatic or mild symptoms with no intervention indicated), moderate AEs (minimal, local, or non-invasive intervention indicated), or severe AEs (disabling; hospitalization, or prolongation of hospitalization indicated; medically significant but not immediately life-threatening).

### Quality of life assessment

The quality of life assessment of the patients was assessed using a validated Patient's Quality of Life Assessment Questionnaire (QoL; refer to the Supplementary Table 5). The questionnaire was filled twice, i.e., once at screening (day 0) and at follow-up (10th day). The QoL questionnaire was divided into three parts, namely, psychology, physiology, and society, and had a total of 20 questions. The score of each section was calculated by the sum of the score of the part divided by the number of questions. The TS was the sum of the three parts, and the minimum importance difference (MID) was set as 1.3, indicating that if the QoL questionnaire score before and after treatment in the same patient raised by 1.3, it indicated that the treatment was effective. The higher the QoL score, the lighter the illness.

### Statistical analysis

The full analysis set (FAS) included subjects as close as possible to the intention-to-treat principle and was used to analyze primary and secondary endpoints. The last observation carried forward (LOCF) method was used to estimate the missing values of the primary endpoints. The per protocol set (PPS) consisted of all the subjects that complied with the study protocol, with drug compliance of 80–120%, and had complete records required in the case report form (CRF). PPS was used for primary and secondary efficacy endpoint analysis. Statistical analysis was performed using SPSS (version 23.0; IBM, NY, USA). Descriptive statistics were reported as proportions, mean ± standard deviation (SD), and median (interquartile range [IQR]) where appropriate. The comparison of continuous variables was made using the *t*-test/rank-sum test and the chi-square test or Fisher's probability exact test was used for the comparison of discrete and categorical variables. All statistical inferences used two-sided tests, with a statistically significant test level of 0.05. Efficacy analysis was applied to both FAS and PPS, while safety analysis was applied to safety set (SS) consisting of all randomized subjects that have used the test drug at least once and have at least one safety assessment record.

## Results

### Patient sample and characteristics

**Figure 1:**
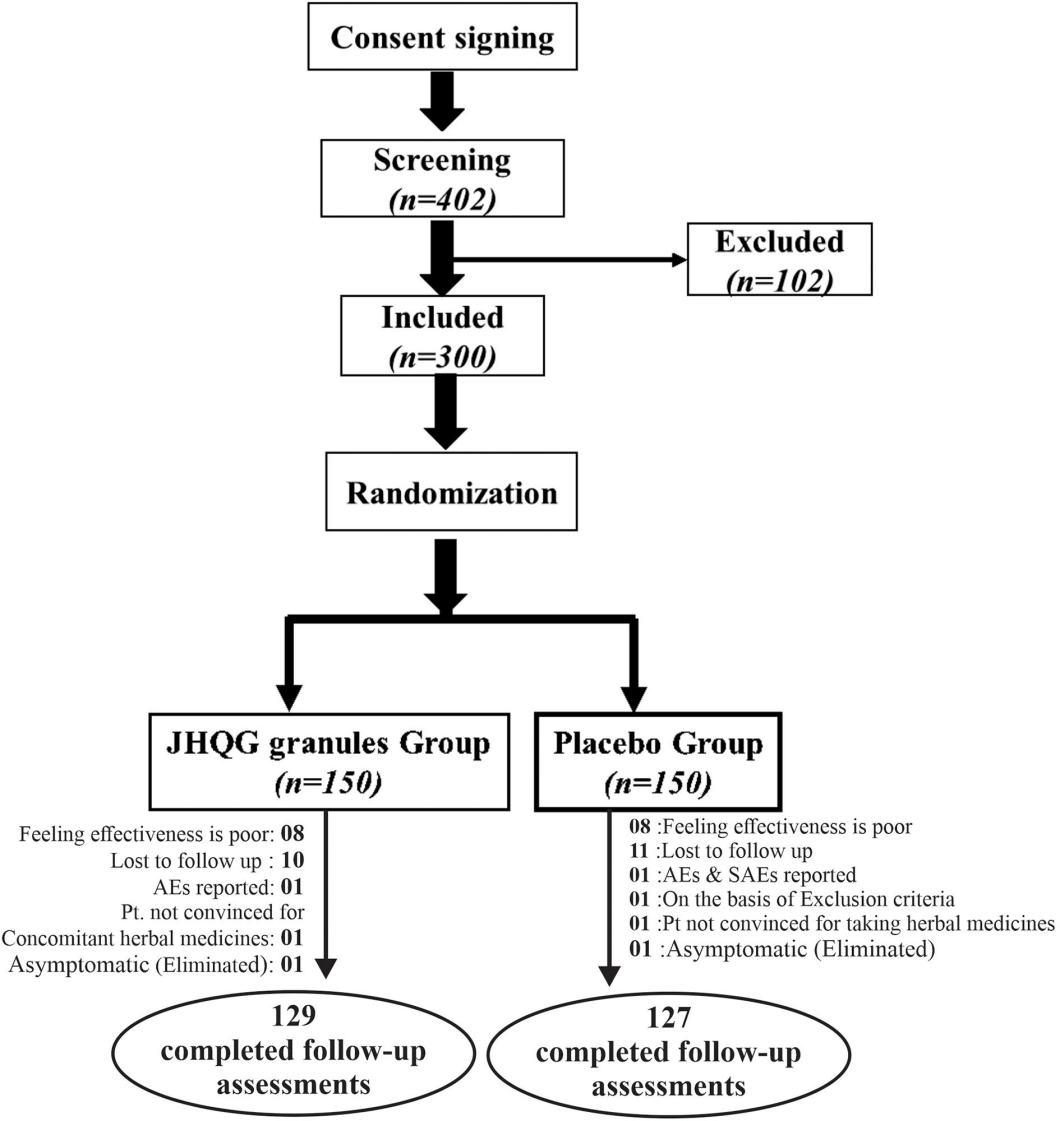
Flowchart of screening, randomization, and treatment of subjects.

A total of 402 RT-PCR-confirmed COVID-19 patients were identified from September 22, 2020 to August 23, 2021, at Indus Hospital (Plot C-76, Korangi Crossing, Karachi, Sindh), Pakistan. Eligible patients (*n* = 300) were recruited and randomly allocated in 1:1 ratio into the JHQG (*n* = 150) and placebo (*n* =150, control) groups, as shown in Figure 1. The enrolled patients were from different ethnicities including 174 Urdu (58%), 12 Pashtuns (4.0%), 6 Baluchis (2.0%), 6 Gilgiti (2.0%), 56 Punjabi (18.66%), 15 Sindhi (5.0%), and 31 others (10.33%). Two patients were eliminated from the study due to concomitant medication use and 42 patients withdrew consent after randomization. Notably, 21 patients in the JHQG group and 23 patients in the placebo group dropped out/withdrew consent from continuing the study. Thus, a total of 256 patients completed the study and were included in the final analysis (PPS analysis). Both groups had no statistical differences in terms of demographic characteristics, concomitant medications, and medical history (Table 1).

**Table 1:** Demographics and baseline characteristics of the subjects in the trial.

**Variable**	**JHQG group (*n* = 150)**	**Placebo group (*n* = 150)**	***P*-value**
**Gender**			0.403
Male (%)	91 (60.67)	98 (65.33)	
Female (%)	59 (39.33)	52 (34.67)	
**Marital status**			
Married (%)	111 (74.00)	108 (72.00)	0.696
Unmarried (%)	39 (26.00)	42 (28.00)	
**Age**			0.517
Mean (SD)	38.89 (11.87)	38.02 (11.47)	
**History of other diseases**		0.529
Yes (%)	26 (17.33)	22 (14.67)	
No (%)	124 (82.67)	128 (85.33)	
**History of drug allergy**			0.556
Yes (%)	5 (3.33)	7 (4.67)	
No (%)	145 (96.67)	143 (95.33)	
**Drug use due to comorbidities or symptoms**			0.607
No (%)	21 (14.00)	18 (12.00)	
Yes (%)	129 (86.00)	132 (88.00)	
**Body temperature (** **°** **C)**			0.771
Mean (SD)	36.92 (0.37)	36.91 (0.38)	
**Respiratory rate (bpm)**			0.693
Mean (SD)	17.50 (1.79)	17.42 (1.72)	
**Pulse (bpm)**			0.475
Mean (SD)	90.67 (12.89)	89.67 (11.27)	
**Systolic blood pressure (mmHg)**			0.140
Mean (SD)	127.45 (14.52)	125.05 (13.53)	
**Diastolic pressure (mmHg)**			0.372
Mean (SD)	80.58 (9.87)	79.58 (9.50)	

### Primary efficacy endpoints

Based on FAS and PPS, each subject's score of MS (i.e., cough and fever) and SS as well as TS were described and compared at baseline and at follow-up for JHQG and placebo groups. The intergroup difference had no statistical significance at baseline (Table 2). Clinical efficacy after treatment was analyzed for each group. FAS showed that the clinical efficacy was 82.67% and 10.74% for the JHQG and placebo groups, respectively, and the rate difference was 71.93% (95% CI 64.09–79.76), indicating that JHQG was superior to placebo (Table 3). The PPS analysis showed that the clinical efficacy was 95.35% and 12.60% for the JHQG and placebo groups, respectively, and the rate difference was 82.75 (95% CI 75.93–89.57; Table 3). Both the FAS and PPS analyses for clinical efficacy comparison between the JHQG and placebo groups showed statistically significant differences (*P* < 0.001 for both), indicating that JHQG was superior to placebo. In FAS, the post-treatment SARS-CoV-2 negative test rate was 38.00% and 42.67% in the JHQG and placebo groups, respectively, with a rate difference of −4.67 (95% CI −15.76 to 6.42). In PPS, the post-treatment SARS-CoV-2 negative test rate was 44.19% and 44.61% in the JHQG and placebo groups, respectively, with a rate difference of −5.42 (95% CI −17.63 to 6.79), and both analyses failed to reach statistically significant outcomes (Table 4).

**Table 2:** Symptom scores (FAS) and (PPS) of the patients.

**Variable**	**JHQG group**	**Placebo group**	***P*-value**
	**(*n* = 150)**	**(*n* = 150)**	
**Symptom score (PPS)**			
**MS score**			0.782
Mean (SD)	2.35 (0.83)	2.32 (0.84)	
Min, Max	0.00, 6.00	0.00, 6.00	
**SS score**			0.848
Mean (SD)	8.49 (4.47)	8.59 (3.94)	
Min, Max	0.00, 22.00	0.00, 18.00	
**TS score**			0.896
Mean (SD)	10.84 (4.65)	10.91 (4.19)	
Min, Max	0.00, 24.00	0.00, 22.00	
**Variable**	**JHQG group**	**Placebo group**	***P*** **-value**
	**(*****n*** = **129)**	**(*****n*** = **127)**	
**Symptom score (PPS)**			
**MS score**			0.579
Mean (SD)	2.31 (0.73)	2.36 (0.77)	
Min, Max	2.00, 4.00	2.00, 4.00	
**SS score**			0.750
Mean (SD)	8.45 (4.34)	8.61 (3.88)	
Min, Max	0.00, 22.00	0.00, 18.00	
**TS score**			0.685
Mean (SD)	10.76 (4.43)	10.98 (4.09)	
Min, Max	2.00, 24.00	2.00, 20.00	

*MS, Main symptoms; SS, Secondary symptoms; TS, Total symptoms*.

**Table 3:** Post-treatment clinical efficacy rate (FAS/PPS), rate difference (FAS/PPS), and inter-group comparison.

**Clinical efficacy**	**FAS**		**PPS**	
	**JHQG group**	**Placebo group**	**JHQG group**	**Placebo group**
**Post-treatment clinical efficacy rate (FAS/PPS)**				
No (ineffective, worsened)	26 (17.33)	133 (89.26)	6 (4.65)	111 (87.40)
Yes (clinically cured, remarkable effective and effective)	124 (82.67)	16 (10.74)	123 (95.35)	16 (12.60)
**Rate difference**	**Test method**	**FAS**	**PPS**
		**Statistics**	**Statistics**
**Clinical efficacy rate difference (FAS/PPS)**			
JHQG group-Placebo group	Rate difference (95% CI)	71.93 (64.09, 79.76)	82.75 (75.93, 89.57)
**Clinical efficacy rate**	**Model**	**FAS**	**PPS**
		***P*** **-value**	***P*** **-value**
**Inter-group comparison (FAS/PPS)**			
JHQG group vs. Placebo group	Chi-square test	<0.0001	<0.0001

**Table 4:** Post-treatment SARS-CoV2 negative test rate (FAS/PPS), test rate difference, and Inter group comparison (FAS/PPS).

**SARS-CoV2 negative test**	**FAS**		**PPS**	
	**JHQG group**	**Placebo group**	**JHQG group**	**Placebo group**
**Post-treatment SARS-CoV2 negative test rate (FAS/PPS)**				
No	93 (62.00)	86 (57.33)	72 (55.81)	64 (50.39)
Yes	57 (38.00)	64 (42.67)	57 (44.19)	63 (49.61)
**SARS-CoV2 rate difference**	**Test method**	**FAS**	**PPS**
		**Statistics**	**Statistics**
**Post-treatment SARS-CoV2 negative test rate difference (FAS/PPS)**			
JHQG group-Placebo group	Rate difference (95% CI)	−4.67 (−15.76, 6.42)	−5.42 (−17.63, 6.79)
**SARS-CoV2 negative test rate**	**Model**	**FAS**		**PPS**	
		**Statistics**	***P*** **-value**	**Statistics**	***P*** **-value**
**Post-treatment SARS-CoV2 negative test rate inter-group comparison**					
JHQG group vs. Placebo group	Chi-square test	0.679	0.410	0.755	0.385

### Secondary efficacy endpoints

The time of defervescence for JHQG was 2.00 days, while it was 2.5 days for placebo with no statistically significant difference. Intergroup comparison of the rate of clinically cured patients, remarkable effective rate, effective rate, ineffective rate, and worsened rate showed statistically significant differences in both FAS and PPS analyses of curative index (*P* < 0.001, Table 5). In the FAS analysis, the rate of clinically cured patients, remarkable effective rate, effective rate, ineffective rate, and worsened rate were 36.66, 22.00, 24.00, 16.67, and 0.67%, respectively, in the JHQG arm, and 1.34, 0.00, 9.39, 82.55, and 6.71%, respectively, in the placebo arm. Intergroup comparisons showed statistically significant results (*P* <0.001). Similarly, in the PPS analysis, the rate of clinically cured patients, remarkable effective rate, effective rate, ineffective rate, and worsened rate were 42.63, 25.58, 27.13, 3.87, and 0.77%, respectively, in the JHQG arm, and 1.57, 0.00, 11.02, 79.52, and 7.87%, respectively, in the placebo arm (*P* < 0.001).

**Table 5:** Curative index analysis (FAS/PPS).

**Variable**	**JHQG group**	**Placebo group**	***P*-value**
**FAS**			<0.001
Worsened (%)	1 (0.67)	10 (6.66)	
Ineffective (%)	25 (16.67)	123 (82.55)	
Effective (%)	36 (24.00)	14 (9.39)	
Remarkable E. (%)	33 (22.00)	0 (0.00)	
Clinically cured (%)	55 (36.66)	2 (1.34)	
**PPS**			<0.001
Worsened (%)	1 (0.77)	10 (7.87)	
Ineffective (%)	5 (3.87)	101 (79.52)	
Effective (%)	35 (27.13)	14 (11.02)	
Remarkable E. (%)	33 (25.58)	0 (0.00)	
Clinically cured (%)	55 (42.63)	2 (1.57)	

The QoL questionnaire values for JHQG and placebo groups' patients at baseline were different, i.e., 8.92 and 9.81 in FAS analysis for JHQG and placebo groups and 8.75 and 9.83 in PPS analysis, respectively. The QoL questionnaire values for JHQG and placebo groups' patients were nearly the same after treatment, i.e., 10.72 and 10.27 in FAS analysis and 10.72 and 10.26 in PPS analysis, respectively. Results of the QoL questionnaire showed that the QoL of patients in the JHQG group improved (1.96 ± 0.75) in comparison with the placebo group (0.44 ± 0.65) after treatment (FAS analysis, *P* < 0.001). Similarly, in PPS analysis, results of the QoL questionnaire showed that the QoL of patients in the JHQG group improved (1.97 ± 0.75) in comparison with the placebo group (0.44 ± 0.65; *P* < 0.001; Table 6).

**Table 6:** Change in quality of life (QoL) questionnaire (FAS/PPS).

**Variable**	**JHQG**	**Placebo**	***P*-value**
	**group**	**group**	
**FAS**			
Before treatment			
*N* (*N* miss)	150 (0)	150 (0)	<0.0001
Mean (SD)	8.9277 (0.9695)	9.8140 (0.8287)	
After-treatment			
*N* (*N* miss)	130 (20)	128 (22)	<0.0001
Mean (SD)	10.7215 (0.6763)	10.2743 (0.8489)	
Before-after treatment			
*N* (*N* miss)	130 (20)	128 (22)	<0.0001
Mean (SD)	1.96 (0.75)	0.44 (0.65)	
**PPS**			
Before treatment			
*N* (*N* miss)	129 (0)	127 (0)	<0.0001
Mean (SD)	8.7574 (0.8556)	9.8334 (0.8568)	
Post-treatment			
*N* (*N* miss)	129 (0)	127 (0)	<0.0001
Mean (SD)	10.7230 (0.6788)	10.2687 (0.8499)	
Before-after treatment			
*N* (*N* miss)	129 (0)	127 (0)	<0.0001
Mean (SD)	1.97 (0.75)	0.44 (0.65)	

The change in radiographic findings in chest X-rays after treatment was also compared between the JHQG and placebo groups. In the FAS analysis, 12.5 and 8.59% of chest X-rays of patients in the JHQG group showed improvement and evidence of worsening after treatment, respectively, while 11.90 and 7.14% showed improvement and evidence of worsening after treatment, respectively, in the placebo group, with no statistically significant differences between the two groups. Similarly, in the PPS analysis, 12.6 and 8.66% of chest X-rays of patients in the JHQG group showed improvement and evidence of worsening after treatment, respectively, while 11.90 and 7.14% showed improvement and evidence of worsening after treatment, respectively, in the placebo group, with no statistically significant differences between the two groups (Table 7).

**Table 7:** Change in radiographic findings of the lungs (FAS/PPS).

**Variable**	**JHQG group**	**Placebo group**	***P*-value**
	***n* (%)**	***n* (%)**	
**FAS**			0.887
Improvement	16 (12.50)	15 (11.90)	
No change	101 (78.91)	102 (80.95)	
Worsened	11 (8.59)	9 (7.14)	
**PPS**			0.887
Improvement	16 (12.60)	15 (12.00)	
No change	100 (78.74)	101 (80.80)	
Worsened	11 (8.66)	9 (7.20)	

The incidence of AEs in the JHQG and placebo groups showed no statistically significant differences (Table 8). In the JHQG group, three patients (2.0%) experienced AEs. In the placebo group, four patients (2.67%) experienced AEs. The placebo group had two patients and 4 incidences of AEs that lead to withdrawal from the study with a 1.33% rate of incidence, while the JHQG group had one patient and two incidences of AEs that lead to withdrawal of consent with a 0.67% rate of incidence.

**Table 8:** Adverse events/reactions and their severity levels observed during the trial (SS).

	**JHQG group**			**Placebo group**		
	**Inc**.	**Sub**.	**Rate (%)**	**Inc**.	**Sub**.	**Rate (%)**
**Adverse events**	**6**	**3**	**2.00**	**8**	**4**	**2.67**
Mild	4	2	1.33	2	1	0.67
Moderate	2	1	0.67	6	3	2.00
Severe	0	0	0.00	0	0	0.00
**Adverse events that lead to withdrawal**	2	1	0.67	4	2	1.33

*Inc., incidences; Sub., subjects*.

The recovery time for the JHQG group from cough, sputum, sore throat, dyspnea, headache, nasal obstruction, fatigue, and myalgia symptoms was shorter than the placebo group (*P* < 0.001). The median time (days) for recovery from cough, sputum, and sore throat symptoms was 6 days (for all) in the JHQG group and more than 11 days in the placebo group. The median time for recovery from fatigue was 7 days in the JHQG group and more than 11 days in the placebo group (*P* < 0.001). There were no statistically significant differences between the two groups in the recovery time from runny nose, chest pain, and diarrhea (Table 9).

**Table 9:** Comparison of the recovery time for individual symptom in JHQG and Placebo group.

**Symptom**	**JHQG group**	**Placebo group**	***P*-value**

	**Median time (days)**	**Median time (days)**	
Cough	6	>11	<0.0001
Sputum	6	>11	<0.0001
Sore throat	6	>11	<0.0001
Dyspnea	5	>11	0.0002
Headache	4	>11	0.0026
Nasal obstruction	4	6	0.0007
Myalgia	4.5	>11	<0.0001
Fatigue	7	>11	<0.0001
Runny nose	2	4	0.0974
Chest pain	4	4	0.3935
Diarrhea	2.5	3	0.4549

The changes in WBC, CRP, and ferritin levels were analyzed before and after treatment. In the FAS analysis, JHQG showed no statistically significant effects on routine blood tests, urinalysis, serum electrolytes, liver function tests (ALT, AST, TBIL, AKP, and γ-GT), renal function tests (BUN and Cr), and ECGs of patients (Table 10).

**Table 10:** Change in WBC, CRP, and Ferritin (FAS/PPS).

**Variable**	**JHQG group**	**Placebo group**	***P*-value**
**Change in WBC, CRP and Ferritin (FAS)**
**WBC**
Before treatment			
*N* (*N* miss)	150 (0)	150 (0)	0.671
Median (Min, Max)	6.70 (2.90, 15.20)	6.75 (3.20, 15.90)	
After treatment			
*N* (*N* miss)	130 (20)	128 (22)	0.633
Median (Min, Max)	7.55 (4.90, 15.60)	7.50 (3.90, 14.20)	
Before—after treatment		
*N* (*N* miss)	130 (20)	128 (22)	0.701
Median (Min, Max)	−0.80 (−10.30, 6.20)	−0.70 (−5.60, 9.50)	
Wilcoxon matching rank-sum test	<0.0001	<0.0001	
			
**CRP**			
Before Treatment			
*N* (*N* miss)	150 (0)	147 (3)	0.573
Median (Min, Max)	2.80 (1.00, 80.30)	2.50 (1.00, 214.0)	
After Treatment			
*N* (*N* miss)	126 (24)	122 (28)	0.813
Median (Min, Max)	2.20 (1.00, 34.50)	2.05 (1.00, 193.8)	
Before-After Treatment		
*N* (*N* miss)	126 (24)	120 (30)	0.913
Median (Min, Max)	0.05 (−33.50, 72.00)	0.00 (−189.60, 206.30)	
Wilcoxon matching rank-sum test	0.044	0.077	
**Ferritin**			
Before Treatment			
*N* (*N* miss)	150 (0)	150 (0)	0.016
Median (Min, Max)	87.40 (2.87, 998.01)	63.85 (4.52, 969.66)	
After-Treatment			
*N* (*N* miss)	130 (20)	128 (22)	0.033
Median (Min, Max)	70.11 (3.84, 302.91)	51.08 (2.21, 345.55)	
Before-After Treatment		
*N* (*N* miss)	130 (20)	128 (22)	0.049
Median (Min, Max)	12.60 (−122.89, 824.63)	4.20 (−304.93, 678.05)	
Wilcoxon matching rank-sum test	<0.0001	<0.0001	
**Change in WBC, CRP, Ferritin (PPS)**
**WBC**			
Before treatment			
*N* (*N* miss)	129 (0)	127 (0)	0.671
Median (Min, Max)	6.80 (3.40, 14.50)	6.70 (3.20, 15.90)	
After treatment			
*N* (*N* miss)	129 (0)	127 (0)	0.633
Median (Min, Max)	7.50 (4.90, 15.60)	7.50 (3.90, 14.20)	
Before-after treatment			
*N* (*N* miss)	129 (0)	127 (0)	0.701
Median (Min, Max)	−0.80 (−10.30, 6.20)	−0.70 (−5.60, 9.50)	
Wilcoxon matching rank-sum test	<0.0001	<0.0001	
**CRP**			
Before treatment			
*N* (*N* miss)	129 (0)	124 (3)	0.573
Median (Min, Max)	2.70 (1.00, 80.30)	2.25 (1.00, 214.00)	
After treatment			
*N* (*N* miss)	125 (4)	121 (6)	0.813
Median (Min, Max)	2.20 (1.00, 34.50)	2.10 (1.00, 193.80)	
Before-after treatment		
*N* (*N* miss)	125 (4)	119 (8)	0.913
Median (Min, Max)	0.10 (−33.50, 72.00)	0.00 (−189.60, 206.30)	
Wilcoxon matching rank-sum test	0.029	0.072	
**Ferritin**			
Before treatment			
*N* (*N* miss)	129 (0)	127 (0)	0.016
Median (Min, Max)	87.85 (2.87, 998.01)	62.98 (4.52, 870.23)	
After treatment			
*N* (*N* miss)	129 (0)	127 (0)	0.033
Median (Min, Max)	69.17 (3.84, 302.91)	51.10 (2.21, 345.55)	
Before-after treatment		
*N* (*N* miss)	129 (0)	127 (0)	0.049
Median (Min, Max)	12.92 (−122.89, 824.63)	4.26 (−304.93, 678.05)	
Wilcoxon matching rank-sum test	<0.0001	<0.0001	

## Discussion

In this study, we demonstrated that JHQG was a safe and effective TCM for the symptomatic relief of COVID-19 patients with mild symptoms. The data showed that JHQG (5 g/sachet) administered orally to patients three times a day for 10 days achieved significant clinical efficacy in the alleviation of COVID-19-related symptoms, reduced post-treatment WBC and acute phase reactant levels, shortened time of recovery for COVID-19-related symptoms, and improved QoL.

The Chinese National Health Commission and State Administration of Traditional Chinese Medicine has recommended JHQG to treat fatigue and fever in patients with COVID-19 during the medical observation period (20). In the 2009 H1N1 flu, studies have shown that the combined treatment of JHQG and oseltamivir can shorten the duration of fever (15, 17). JHQG has shown a significant effect on treating mild COVID-19-related symptoms by shortening the period of fever, reducing inflammation, and improving symptoms (16, 18, 21). JHQG is synthesized from Maxingshigan Decoction and Yinqiao Powder, and has the effects of “soothing wind,” ventilating the lungs and clearing away heat and toxic materials (18).

A total of 256 patients, including 129 patients in the JHQG group and 127 patients in the placebo group, completed the study. A total of 44 subjects dropped out (21 in the JHQG group and 23 in the placebo group), causing a 14.66% dropout rate. The basic reason for dropout was that the subjects were unable or unwilling to continue the clinical trial and voluntarily requested to withdraw. The JHQG group showed greater clinical efficacy (82.67%) on the 10th day of treatment compared with placebo (10.74%). The recovery time for the JHQG group from cough, sputum, sore throat, dyspnea, headache, nasal obstruction, fatigue, and myalgia symptoms was shorter as compared to the placebo group (6 vs. >11 days; *P* < 0.05). A previous study has reported that JHQG could shorten the duration of fever, reduce the use of antibiotics, and alleviate respiratory symptoms in patients with influenza A H1N1 (15). In a recent RCT, JHQG combined with western medicine relieved the clinical symptoms of fever and poor appetite in patients with COVID-19 and reduced the use of antibiotics to a certain extent (18). It was suggested that certain compounds in JHQG could bind to specific target proteins and inhibit the activity of SARS-CoV-2 as revealed by high-throughput molecular docking and network pharmacology studies (22, 23). Various active ingredients in JHQG, including quercetin and kaempferol, are hypothesized to target AEC2 and 3CL protein, inhibiting inflammatory mediators, eliminating free radicals, and regulating immunity (24). These proposed mechanisms could explain the shortened recovery time of COVID-19 symptoms secondary to the host inflammatory response after infection.

The post-treatment SARS-CoV2 negative test rate for JHQG and placebo groups was 38.00 and 42.67%, respectively. There was no statistically significant difference in the SARS-CoV2 negative test rate in both groups. No clear benefit is shown in terms of viral clearance in both groups; however, the negative test rate in the JHQG group was higher (38%) than in a previous study (8.3%) (18). One reason behind this could be the shorter time (i.e., 10th day) to assess the negative SARS-CoV2 rate. Various clinical trials on patients with COVID-19 used a 28-day time interval for the assessment of negative SARS-CoV2 test rate. However, even no statistically significant difference has been reported for negative SARS-CoV2 patients after 28 days among patients receiving remdesivir and those receiving placebo (25). In addition, JHQG has immunomodulatory activity against COVID infection, which can explain the clinical efficacy. The PCR test detects the presence of viral RNA. This does not necessarily imply that the viral RNA is totally active. The main chemical constituents of JHQG explored *via* the modern pharmacological approach include stigmasterol, kaempferol, and quercetin, which possess anti-inflammatory, immunomodulatory, and antiviral effects (26). In total, 10 AEs/reactions were observed during the trial. Overall, JHQG was well-tolerated and only three patients in the treatment group experienced mild-to-moderate AEs. JHQG also showed no clinically significant effects on routine blood tests, urinalysis, serum electrolytes, liver function tests, renal function tests (BUN and Cr), and ECGs. These findings provide evidence to support that JHQG is a safe TCM among mild COVID-19 infection cases.

To the best of our knowledge, this is the first RCT to evaluate the clinical efficacy and safety of JHQG in the treatment of laboratory-confirmed non-hospitalized COVID-19 patients. Various limitations of this trial should be noted. The basic reason for dropout was that the subjects were unable or unwilling to continue the clinical trial and voluntarily requested to withdraw. The study included only patients with COVID-19 of Pakistani race, which may limit the geographic generalizability of the findings. This study also excluded patients with severe underlying medical conditions, who are at particularly heightened risk of COVID-19 disease progression. Future studies of JHQG in COVID-19 shall focus on evaluating the clinical efficacy and safety of this TCM in such a group of patients. In conclusion, our data show that JHQG is a safe and effective treatment for COVID-19 patients with mild symptoms.

## Data availability statement

The raw data supporting the conclusions of this article will be made available by the authors, without undue reservation.

## Ethics statement

The studies involving human participants were reviewed and approved by Institutional Ethics Committee of the International Center for Chemical and Biological Sciences (ICCBS), University of Karachi, Institutional Review Board of the Indus hospital (Sector 39, Karachi, Sindh, Pakistan) and National Bioethics Committee (NBC) Pakistan. The patients/participants provided their written informed consent to participate in this study.

## Author contributions

MS: principal investigator of the trial and trial management. SF and SK: clinical research coordination. SU: management of trial and regulatory affairs. GH: clinical investigator. KW: initial draft preparation. TL: data curation. JL: reviewing the data. QL: reviewing the manuscript. DL: finalized the data and manuscript. All authors contributed to the article and approved the submitted version.

## Funding

The authors declare that this study received funding from Ju Xie Chang (Beijing) Pharmaceuticals Co., Ltd., Beijing, China. The funder was not involved in the study design, collection, analysis, interpretation of data, the writing of this article, and the decision to submit it for publication.

## Conflict of interest

The authors declare that the research was conducted in the absence of any commercial or financial relationships that could be construed as a potential conflict of interest.

## Publisher's note

All claims expressed in this article are solely those of the authors and do not necessarily represent those of their affiliated organizations, or those of the publisher, the editors and the reviewers. Any product that may be evaluated in this article, or claim that may be made by its manufacturer, is not guaranteed or endorsed by the publisher.
